# MYL6B drives the capabilities of proliferation, invasion, and migration in rectal adenocarcinoma through the EMT process

**DOI:** 10.1515/biol-2020-0031

**Published:** 2020-08-03

**Authors:** Jin-Liang Li, Zai-Qiu Wang, Xiao-Li Sun

**Affiliations:** Department of Anus & Intestine Surgery, The First People's Hospital of Jining, Jining, Shandong, 272100, P.R. China; Department of Anorectal Surgery, Yantai Yuhuangding Hospital, Yantai, 264000, P.R. China; Department of Clinical Laboratory, Yantai Yuhuangding Hospital, Yantai, 264000, P.R. China

**Keywords:** MYL6B, rectal adenocarcinoma, prognosis, EMT markers

## Abstract

**Objective:**

This study was designed to explore the biological significance of myosin light chain 6B (MYL6B) in rectal adenocarcinoma.

**Methods:**

Profiles on the Oncomine dataset, GEPIA website, and UALCAN-TCGA database were searched to assess the MYL6B expression level in rectal adenocarcinoma tissues and normal tissues. After MYL6B knockdown using siRNA strategy, cell counting kit-8 (CCK-8) and transwell assays were conducted to measure cell proliferation, migration and invasion, respectively. Flow cytometry analysis was conducted to assess cell apoptosis. Quantitative reverse transcription-polymerase chain reaction (qRT-PCR) and western blot were performed to detect the expression level of mRNAs and proteins.

**Results:**

The data showed that overexpression of MYL6B was observed in rectal adenocarcinoma tissues and correlated with a poor prognosis of patients. Functional *in vitro* experiments revealed that MYL6B knockdown could inhibit proliferation, migration, and invasion of rectal adenocarcinoma cells, while promote cell apoptosis. Moreover, western blot analysis suggested that increased expression of E-cadherin and decreased expression of N-cadherin and Vimentin were induced by si-MYL6B.

**Conclusion:**

In summary, this study elaborated on the promoting effect of MYL6B in rectal adenocarcinoma progression, thus providing novel insight for strategies of clinical diagnosis and drug application in the future clinical study.

## 1 Introduction

Rectal cancer is a leading cause of cancer-related mortality with a total of 140,250 new cases in 2018, which accounts for 8.1% of all new cancer cases [1]. As a kind of rectal cancer, rectal adenocarcinoma plays a major role due to its high incidence and mortality [2,3]. The incidence of rectal adenocarcinoma has been reported to be increasing year by year in many Asian countries [4]. Despite significant advances in screening and surgery with chemotherapy, overall survival rates for patients diagnosed with advanced rectal adenocarcinoma remain low [5]. The outcomes of patients with rectal adenocarcinoma rely on the time of diagnosis, and targeted treatment is beneficial for this [6,7,8]. However, targeted treatment of rectal adenocarcinoma still has a relatively minor exploration.

Myosin is a superfamily of motor proteins, which is well known for its pronounced effects on motility processes, and a myosin light chain could modulate the Ca^2+^ transduction [9,10]. As an essential myosin light chain, myosin light chain 6B (MYL6B) is involved in cell viability, adhesion, migration and endocytosis, tissue architecture, and cargo transport [11,12,13]. Recently, increasing attention has been paid to the role of MYL6B in the progression of various cancers, including colon cancer and hepatocellular carcinoma [14,15,16]. However, the function of MYL6B in rectal adenocarcinoma has not yet been determined.

Therefore, in the present study, the expression level and prognostic significance of MYL6B in rectal adenocarcinoma were analyzed. Based on these analyses, the proliferation, migration, and invasion of rectal adenocarcinoma cells after MYL6B knockdown *in vitro* were evaluated. Subsequently, the expression levels of epithelial–mesenchymal transition (EMT)-related markers such as E-cadherin, N-cadherin, and Vimentin were examined by western blot analysis. This study illustrated the role of MYL6B in rectal adenocarcinoma, suggesting a potential therapeutic target for its progression and diagnosis.

## 2 Materials and methods

### 2.1 Clinical data analysis of rectal adenocarcinoma

Data on MYL6B expression in the 65 normal rectum and 65 rectal adenocarcinoma tissue samples were obtained from the Oncomine dataset. In addition, a comparative analysis of the MYL6B expression in 92 human normal and 10 tumor specimens was acquired from the GEPIA website. Based on these data, the overall survival was evaluated.

### 2.2 Cell culture

Human rectal adenocarcinoma cell lines (HT-29, SW1116, HCT116, and SW480) and normal control cell line Hs680.Rec were purchased from the Shanghai Cell Bank of the Chinese Academy of Medical Sciences (Shanghai, China). All the cells were incubated in the RPMI-1640 culture medium supplemented with 10% fetal bovine serum, 1% penicillin and streptomycin at 37°C and 5% CO_2_. When the cells entered the logarithmic growth stage, digestion was used to round the cells after washing with phosphate buffered saline (PBS) for three times. Finally, the cells were seeded into a six-well plate for subsequent experiments.

### 2.3 Quantitative reverse transcription-polymerase chain reaction (qRT-PCR)

According to the manufacturer’s protocols, total RNA was collected from cells by using TRIzol reagent (Invitrogen, Carlsbad, CA, USA), and then cDNA was generated using SuperScript III reverse transcriptase (Invitrogen, Carlsbad, CA, USA). The amplification was implemented with an Applied Biosystems 7500 Real-Time PCR System (Thermo Fisher Scientific, Inc., Carlsbad, CA, USA): 95°C for 5 min, 95°C for 30 s with 40 cycles, 60°C for 45 s, and 72°C for 30 min. The sequences of the primers were as follows: MYL6B: F 5′-ACTTGGAGGGGTTTCGTGTG-3′, R 5′-CGTAGTTGATGCAGCCGTTG-3′; actin: F 5′-CCCGAGCCGTGTTTCCT-3′, R 5′-GTCCCAGTTGGTGACGATGC-3′. For each sample, the process was repeated three times, and the expression of MYL6B was calculated by the 2^−△△Ct^ method.

### 2.4 Cell transfection

For cell transfection, cells entering the logarithmic growth phase were placed in a six-well plate with fresh medium and transfected using Lipofectamine 2000 transfection reagent (Thermo Fisher Scientific, Inc., Carlsbad, CA, USA). After transfection for 24 h, the transfection efficiency of siRNAs was detected. The sequences of siRNAs were designed as follows: si-MYL6B#1: 5′-CCTCGATCCCCGATAG-3′, si-MYL6B#2: 5′-GTGTTTGACAAAGAGGGC-3′, and si-con: 5′-AATTCTCCGAACGTGTCACGT-3′.

### 2.5 Cell proliferation assay

Cell proliferation was explored directly by the cell counting kit-8 (CCK-8) assay. The transfected cells were suspended, and then placed into the 96-well plate at a density of 1,000 cells per well. Subsequently, 10 μL of CCK-8 reagent was added into each well before detecting cell viability at 24, 48, and 72 h. The absorbance values were measured at a wavelength of 450 nm with a spectrometer (Multimode Reader; PerkinElmer, USA).

### 2.6 Cell migration and invasion assays

The investigation of cell migration and invasion was conducted using transwell chambers with an 8 μm pore size polycarbonate membrane (Costar, Corning, NY, USA). To detect the migratory ability, 5,000 cells were suspended in 100 μL of medium and then cultured in the upper chamber. The lower chamber was filled with 500 μL of medium. After maintaining at 37°C with 5% CO_2_ overnight, nonmigrating cells were removed using cotton swabs. Next, migrating cells were fixed with 4% paraformaldehyde for 30 min and stained with 0.1% crystal violet for 20 min. Five fields were selected randomly, imaged, and analyzed statistically. To examine the invasive ability, the upper chamber was precoated with Matrigel (BD Biosciences, Shanghai, China), and the inoculation density of cell suspension was 1 × 10^5^ per well. The rest of the steps were the same as for the migration assay.

### 2.7 Cell apoptosis analysis

Cell apoptosis was assessed by Annexin-V kit (70-AP101-100-AVF; MultiSciences, China). After transfection, the cells were washed with pre-cold PBS and then resuspended in 300 μL of binding buffer. Afterward, 5 μL of Annexin-V/FITC reagent was added to the cells and incubated for 15 min in the dark. Subsequently, 5 μL of propidium iodide was added to stain the cells for 5 min. Finally, after adding 200 μL of binding buffer, the cell apoptosis was examined using a flow cytometer (BD Biosciences, USA).

### 2.8 Western blot analysis

After 48 h of transfection, total proteins were extracted from cells with RIPA (with protease inhibitors) and the concentrations of proteins were determined by the bicinchoninic acid method. The proteins were boiled with 5× loading buffer at 95°C for 5 min, separated with 12% SDS-polyacrylamide gel, and then transferred onto polyvinylidene fluoride (PVDF) membrane (Bio-Rad Co., USA). Then the membrane was blocked in Tris-buffered saline containing 0.1% Tween 20 (TBST) that was supplemented with 5% skimmed milk for 1 h at room temperature and incubated with primary antibody (1:1,000; Cell Signaling Technology, Danvers, MA, USA) at 4°C overnight against E-cadherin (cat14472), N-cadherin (cat14215), Vimentin (cat49636), or GAPDH (cat51332). After washing with TBST in triplicates, the PVDF membrane was incubated with secondary antibody (1:2,000; Cell Signaling Technology, Danvers, MA, USA) for 1 h. The protein blots were visualized by enhanced chemiluminescence (ECL) reagent, and the bands were quantified through Bio ID software.

### 2.9 Statistical analysis

Data were analyzed using SPSS 22.0 statistics software and presented as mean ± standard deviation. Differences between two groups were assessed by Student’s *t*-test, and one-way analysis of variance followed by Dunnett’s *post hoc* test was used to describe the mean comparisons in multiple groups. The overall survival was exhibited by the Kaplan–Meier method. The differences were considered statistically significant at *P* < 0.05.

## 3 Results

### 3.1 MYL6B was upregulated and associated with poor prognosis in patients with rectal adenocarcinoma

**Figure 1:**
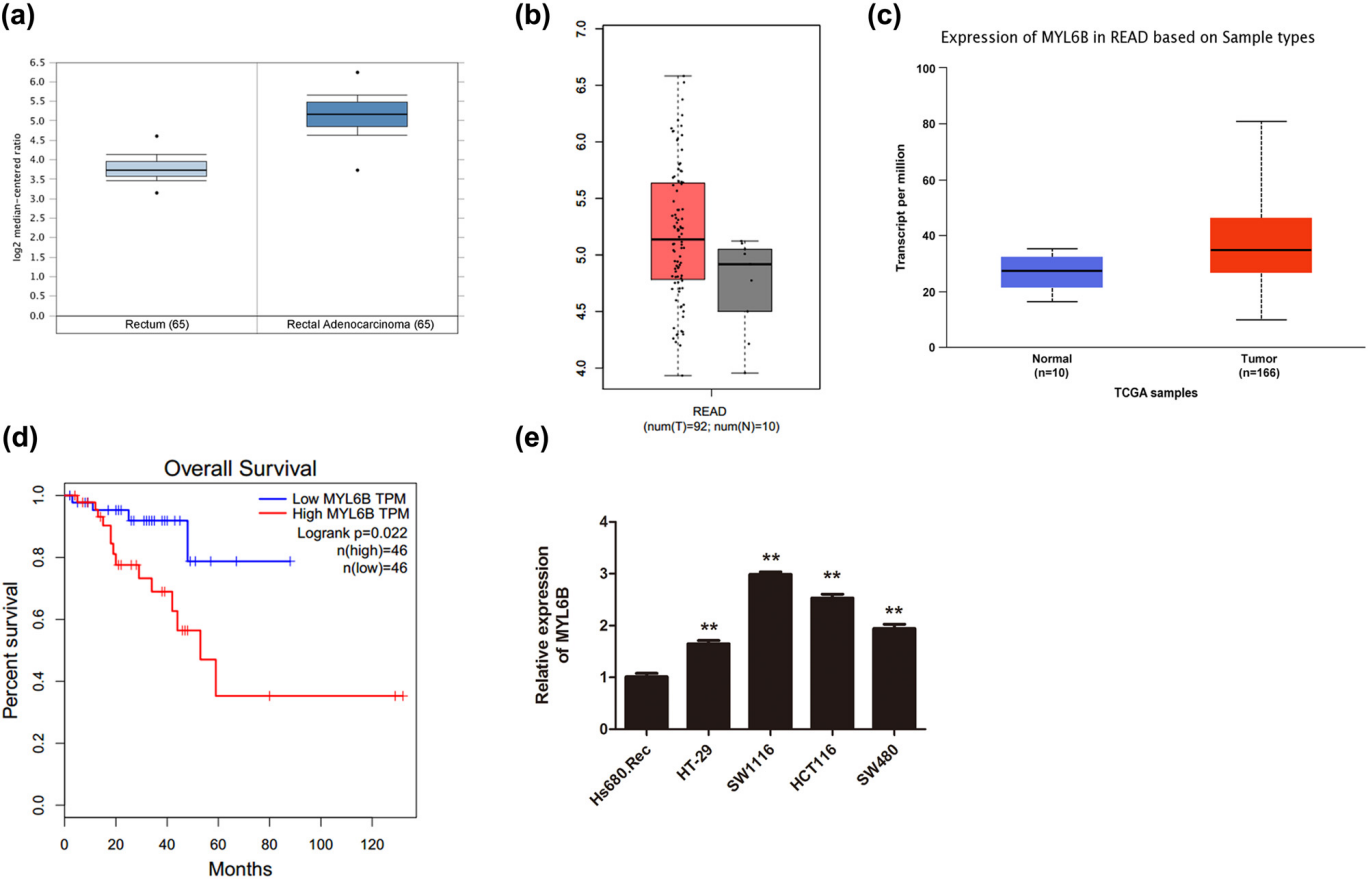
MYL6B expression is upregulated in rectal adenocarcinoma and its overexpression could shorten survival of patients with rectal adenocarcinoma. (a) The mRNA levels of MYL6B in 65 cases of normal rectum and 65 cases of rectal adenocarcinoma, *P* = 1.75 × 10^−40^. (b) The expressional analysis of MYL6B in rectal adenocarcinoma obtained from the GEPIA website, *P* < 0.01. (c) UALCAN-TCGA database analysis of the MYL6B expression in rectal adenocarcinoma based on sample types (10 normal cases and 166 tumor samples), *P* < 0.01. (d) The comparison of overall survival between the groups with high and low expression of MYL6B, *P* = 0.022. (e) Quantitative analysis of MYL6B expression levels in different cell lines, **P < 0.01.

**Figure A1:**
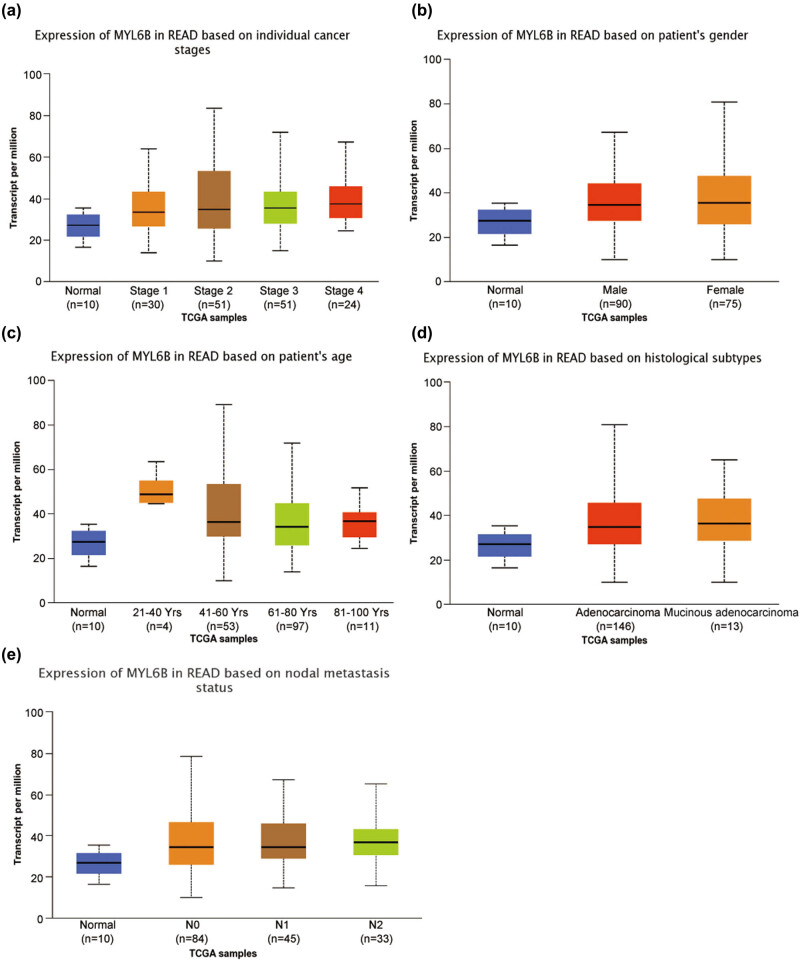
Expression of MYL6B was determined using UALCAN-TCGA database based on (a) individual cancer stages, (b) gender, (c) age, (d) histological subtypes, and (e) nodal metastasis status.

The data obtained from the Oncomine dataset, GEPIA website, and UALCAN-TCGA database showed that the MYL6B expression in rectal adenocarcinoma was higher than that in human normal tissues (Figure 1a–c, *P* < 0.01). Additionally, the MYL6B expression in rectal adenocarcinoma based on individual cancer stages, gender, age, histological subtypes, and nodal metastasis status also revealed statistical significance (Figure A1). In order to determine the role of MYL6B in rectal adenocarcinoma, the overall survival curve was constructed by the Kaplan–Meier method, indicating that upregulation of MYL6B in patients with rectal adenocarcinoma significantly correlated with poor prognosis (Figure 1d, *P* = 0.022). Moreover, the qRT-PCR analysis demonstrated that MYL6B was also upregulated in human rectal adenocarcinoma cell lines (HT-29, SW1116, HCT116, and SW480) at different degrees when compared with that of human normal cell line Hs680.Rec (Figure 1e, *P* < 0.01). These findings suggested that MYL6B might play a crucial role in the development of rectal adenocarcinoma as a carcinogenic factor.

### 3.2 Detection of MYL6B knockdown efficiency

**Figure 2:**
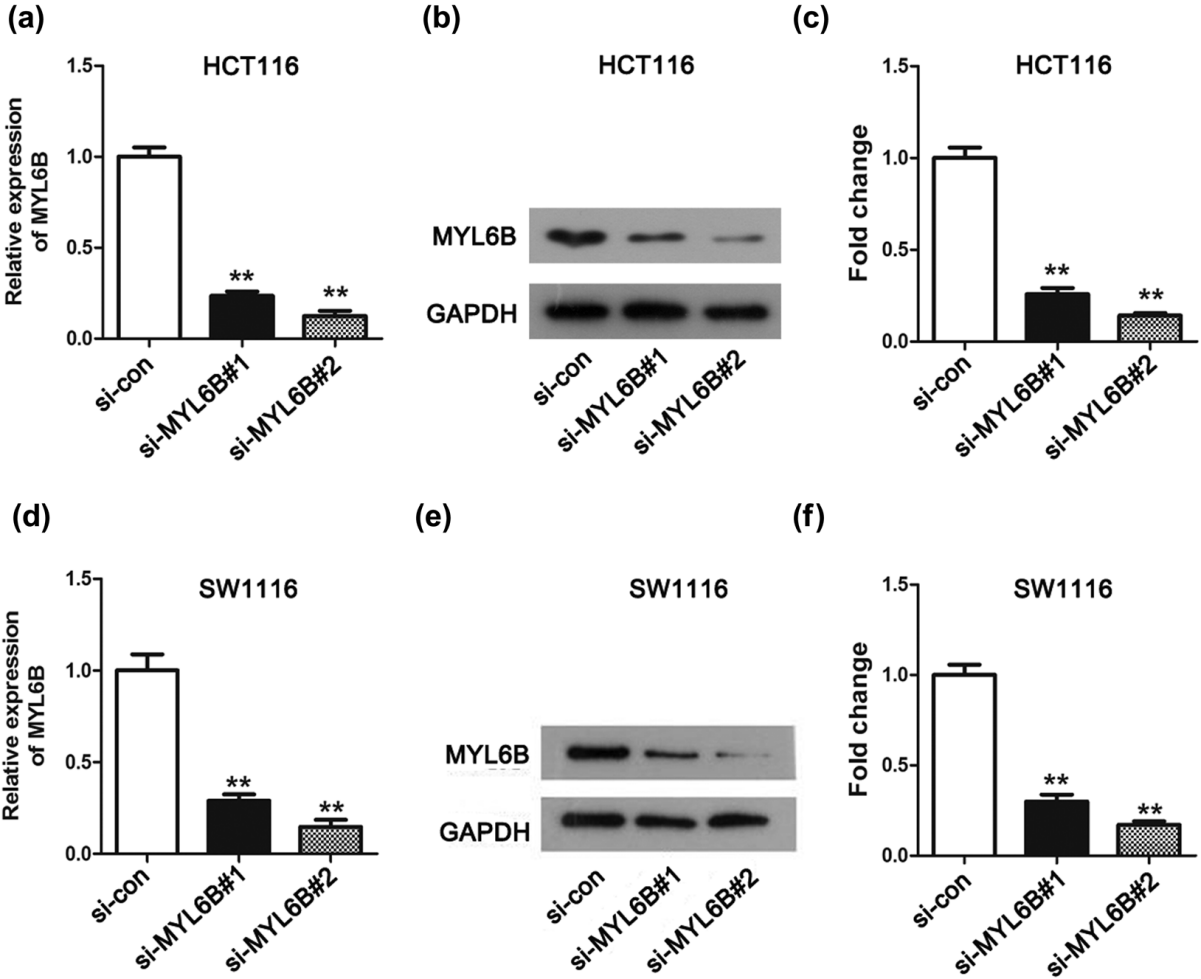
Detection of MYL6B knockdown efficiency in HCT116 and SW1116 cells. The interference of si-MYL6B reduced the MYL6B expression confirmed by the qRT-PCR method in (a) HCT116 and (d) SW1116 cells, **P < 0.01. The investigation of MYL6B knockdown efficiency through western blot analysis and quantification in (b and c) HCT116 and (e and f) SW1116 cells, **P < 0.01.

Next, we examined the biological functions of MYL6B in rectal adenocarcinoma cells after MYL6B knockdown. Considering the relative expression of MYL6B in different cell lines, we selected the HCT116 and SW1116 cell lines as experimental materials due to the higher MYL6B expression level in comparison to the remaining rectal adenocarcinoma cell lines HT-29 and SW480 (Figure 1e, *P* < 0.01). The MYL6B expression level was confirmed by qRT-PCR and western blotting. As shown in Figure 2a and d, MYL6B mRNA expression was significantly disturbed after si-MYL6B#1 and si-MYL6B#2 transfection in HCT116 and SW1116 cells, especially si-MYL6B#2 (*P* < 0.01). Similarly, the same tendency was also exhibited with respect to protein in HCT116 and SW1116 cells (Figure 2b and e). The intensity of protein bands also exhibited a declining pattern when compared with the si-con group (Figure 2c and f, *P* < 0.01). Given the higher knockdown efficiency of si-MYL6B#2, we chose si-MYL6B#2 in further experiments.

### 3.3 Knockdown of MYL6B inhibited proliferation, migration, and invasion while induced apoptosis of rectal adenocarcinoma cells *in vitro*

**Figure 3:**
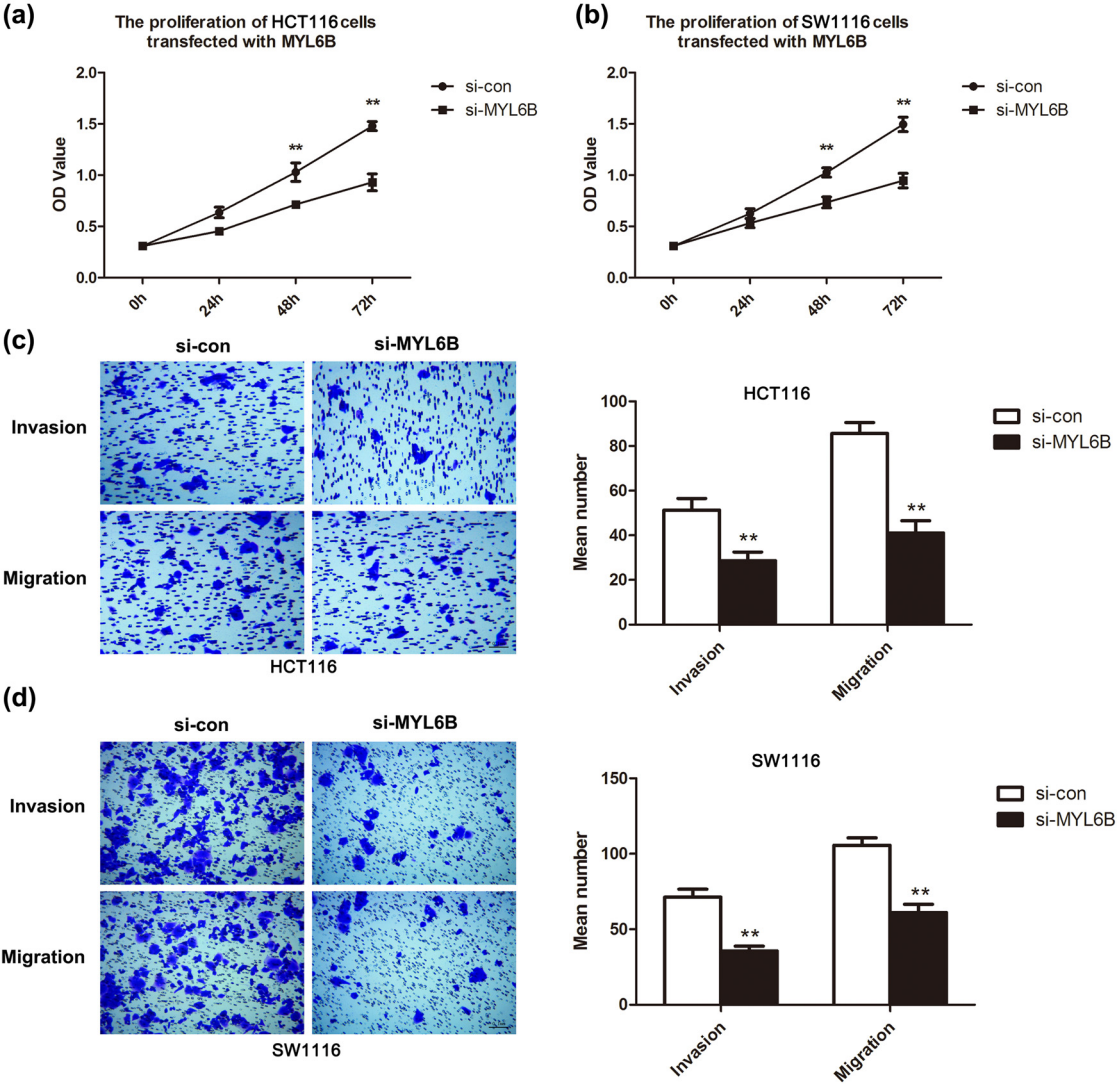
MYL6B knockdown suppressed cell proliferation, migration, and invasion of HCT116 and SW1116. (a and b) Cell proliferation was assessed by CCK-8 assay, **P < 0.01. (c and d) Results of transwell assays identified that cell migratory and invasive capabilities were blocked due to MYL6B knockdown, bar = 200 µm. The invasive and migratory cell numbers were quantified, **P < 0.01.

**Figure 4:**
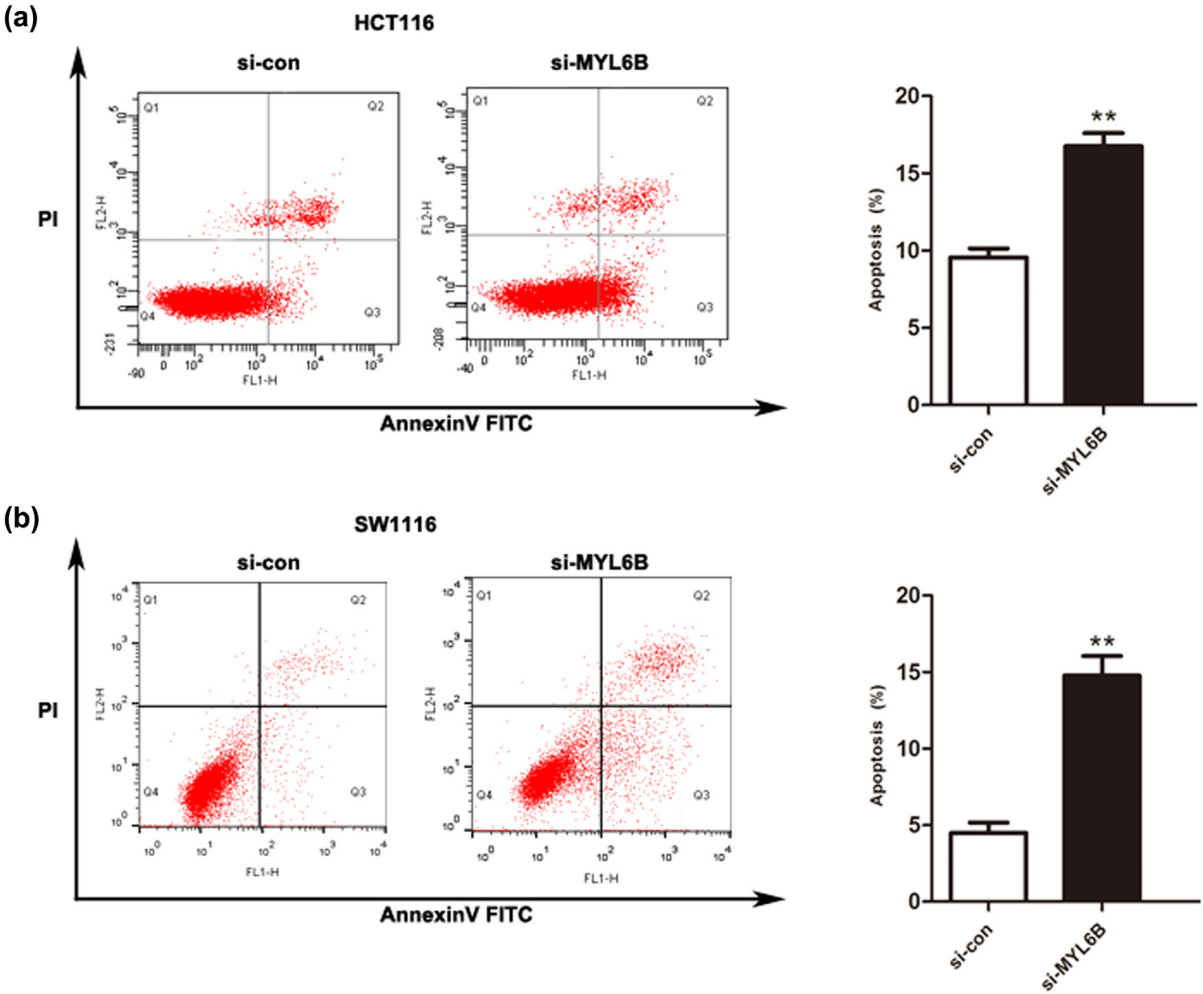
Downregulation of MYL6B induced cell apoptosis. Flow cytometry analyses were performed to assess the apoptotic ability of (a) HCT116 and (b) SW1116 cells, **P < 0.01.

Based on downregulated MYL6B, the proliferation of HCT116 and SW1116 cells was suppressed remarkably at 48 and 72 h (Figure 3a and b, *P* < 0.01). We further performed transwell assays to determine cell migratory and invasive abilities. The representative images in Figure 3c and d indicate that knockdown of MYL6B retarded cell migration and invasion when compared with that of si-con groups. Moreover, the quantitative analysis confirmed the inhibitory effect as the number of migratory and invasive cells decreased significantly when compared with that of the si-con group (Figure 3c and 3d, *P* < 0.01). Results of flow cytometry analysis in Figure 4a and b show that MYL6B inhibition remarkably promoted cell apoptosis in both HCT116 and SW1116 cells (*P* < 0.01). These *in vitro* results aligned with the finding that upregulated MYL6B is accompanied by poor prognosis in patients with rectal adenocarcinoma.

### 3.4 Downregulation of MYL6B suppressed cell malignant behaviors via mediating the EMT process in rectal adenocarcinoma

**Figure 5:**
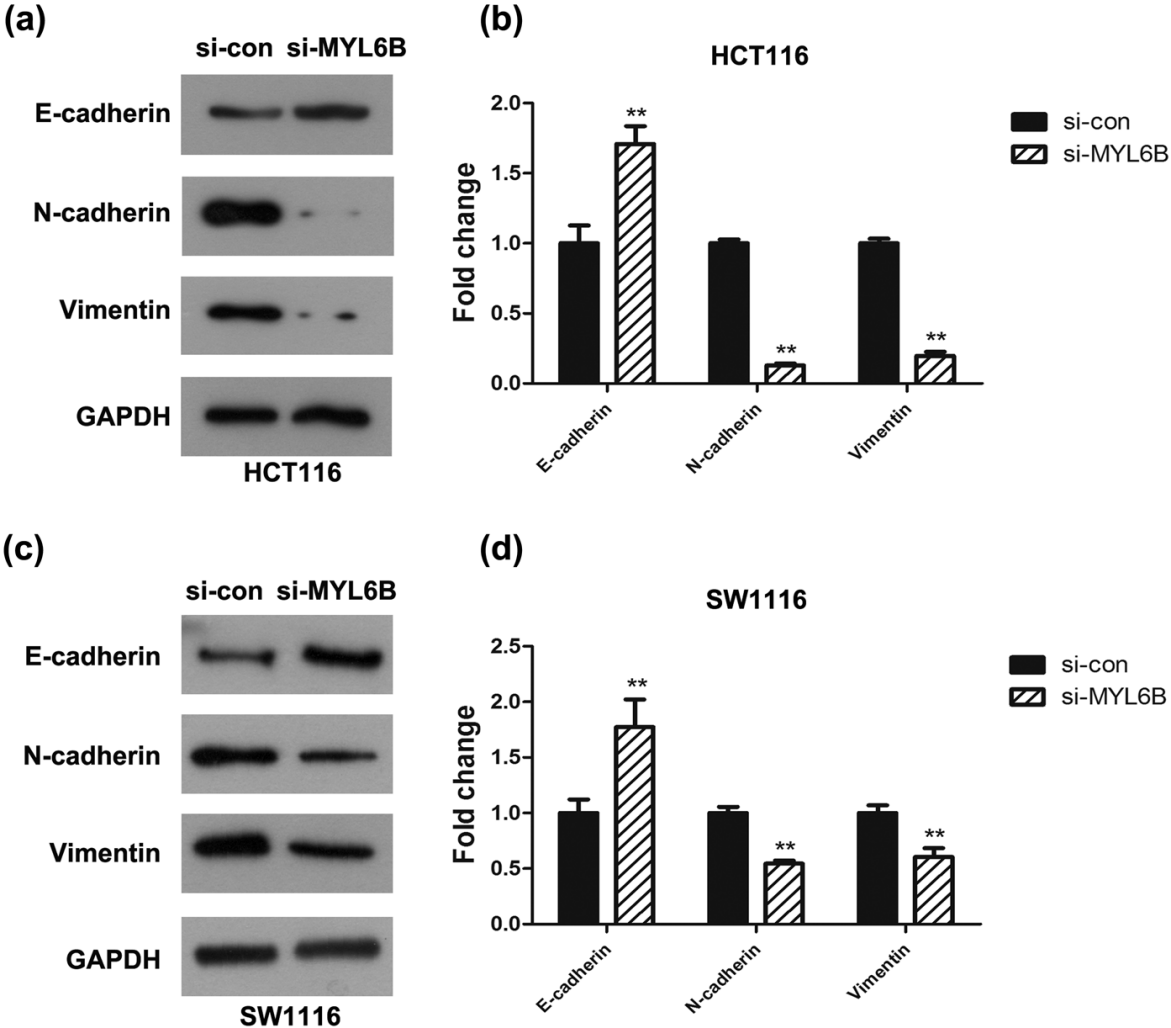
Downregulation of MYL6B suppressed cell malignant behaviors via mediating the EMT process in rectal adenocarcinoma. Western blot results showed that E-cadherin expression was upregulated, while N-cadherin and Vimentin expression was attenuated in (a and b) HCT116 and (c and d) SW1116 cells after MYL6B knockdown, **P < 0.01.

EMT is a process by which epithelial cells become mesenchymal stem cells when they lose their cell polarity and adhesion and gain migratory and invasive properties [17]. This highlighted the crucial role of EMT in tumorigenesis [18]. In accordance with this notion and our aforementioned results, western blotting was conducted to measure the key markers of EMT, including E-cadherin, N-cadherin, and Vimentin expression levels in HCT116 and SW1116 cells that were transfected with si-MYL6B. Upon comparing with the si-con group, we found that MYL6B knockdown led to the upregulation of E-cadherin and the downregulation of N-cadherin and Vimentin (Figure 5a and c). Meanwhile, the fold change also revealed that the reduction of MYL6B hindered N-cadherin and Vimentin but stimulated E-cadherin (Figure 5b and d, *P* < 0.01). These findings suggested that the EMT process is involved in the inhibitory role of MYL6B reduction in rectal adenocarcinoma cells.

## 4 Discussion

Based on the data obtained from the Oncomine dataset, GEPIA website, and UALCAN-TCGA database, this study confirmed the expression level of MYL6B in tumor specimens and cell lines. It revealed that MYL6B may be a new prognostic marker in rectal adenocarcinoma. The overexpression of MYL6B closely correlated with the worse survival of patients with rectal adenocarcinoma. Moreover, MYL6B knockdown resulted in a decreased tendency with respect to biological features, such as proliferation, migration, and invasion. These findings demonstrated that MYL6B is an important biomarker in the development of rectal adenocarcinoma.

Although little is known about the biological function of MYL6B, myosin holoenzymes have been relatively well described. For instance, myosin VI is regulated by DNA damage in a p53-dependent manner and possesses a promoted effect in the p53-dependent pro-survival pathway in RKO cells [19]; disordered expression of myosin-X could cause poor prognosis in breast cancer [20]; and Myh9 could stimulate the development of squamous cell carcinomas [21]. In addition, myosin is composed of heavy chains and light chains, which are important for biological process regulation [22]. Several reports have previously pointed out that myosin light chain proteins were involved in a number of diseases. Huang and Szczesna-Cordary found that hypertrophic, dilated cardiomyopathy and sudden cardiac death are related to myosin light chain [23]. The mutations of N- and C-terminal in MYL2 and MYL3 were linked with these diseases [24,25]. With respect to inflammation, MYL9, MYL12a, and MYL12b have been determined as new copartners with CD69 [26,27]. As an essential light chain for nonmuscle Myosin II (NMII), MYL6B has key impacts on hepatocellular carcinoma by binding to MDM2 [14]. Our findings in this study illustrated that the reduction of MYL6B hampered cell proliferation, migration, and invasion, which is consistent with the aforementioned data about the poor outcome in patients with rectal adenocarcinoma.

EMT plays a very important role in various cancers, of which rectal adenocarcinoma is most prominent [17,18,28]. Moreover, EMT has been reported to act as a vital process in the early metastatic dissemination of tumor cells via endowing them with a more invasive phenotype [29]. Given the inhibitory effect of MYL6B knockdown on invasion and migration in rectal adenocarcinoma cells, we hypothesized that this inhibition is regulated through the EMT pathway. Thus, the expression level of EMT markers was probed to identify the carcinogenic role of MYL6B: E-cadherin was upregulated while N-cadherin and Vimentin were downregulated. Taken together, all the results determined that MYL6B is a putative driver gene in rectal adenocarcinoma, and extensive research should be performed in the future to detect a potential mechanism through *in vivo* experiments to verify the results obtained in this study.

In total, we observed that overexpression of MYL6B could result in poor prognosis in patients with rectal adenocarcinoma, and cell viability, invasion, and migration were suppressed because of MYL6B knockdown. Moreover, the reduction of MYL6B could promote cell apoptosis. Meanwhile, the EMT process was also hindered in rectal adenocarcinoma cells after transfection with si-MYL6B. These findings demonstrated that MYL6B may be a potential prognostic biomarker and therapeutic target in rectal adenocarcinoma, increasing the possibility of cancer cure.
