# Erratum to “MYL6B drives the capabilities of proliferation, invasion, and migration in rectal adenocarcinoma through the EMT process”

**DOI:** 10.1515/biol-2020-0103

**Published:** 2020-10-28

**Authors:** Zai-Qiu Wang, Xiao-Li Sun, Jin-Liang Li

In the published article “Li J-L, Wang Z-Q, Sun X-L. MYL6B drives the capabilities of proliferation, invasion, and migration in rectal adenocarcinoma through the EMT process. Open Life Sci. 2020;15:522–31. doi: 10.1515/biol-2020-0031” the order of authors is incorrect.

The correct order of authors is as follows: Zai-Qiu Wang^#^, Xiao-Li Sun^#^, Jin-Liang Li*

^#^ These authors contributed equally to this work

* Corresponding author

